# Gut microbiome dysbiosis in PCOS: from pathogenesis to microbiome-targeted therapies

**DOI:** 10.3389/fendo.2026.1747766

**Published:** 2026-04-13

**Authors:** Xinyun Du, Hao Su, Yuexi Huang, Jiani Liu, Qiaoying Li, Xuping Yang, Xuemei Tao, Rong Li

**Affiliations:** 1 Department of Pharmacy, Luzhou Maternal and Child Health Hospital, Luzhou Second People’s Hospital, Luzhou Women and Children’s Hospital, Luzhou, China; 2 School of Clinical Medicine, Southwest Medical University, Luzhou, China; 3 School of Pharmacy, Southwest Medical University; Department of Pharmacy, The Affiliated Hospital of Southwest Medical University, Luzhou, China; 4 Department of Nephrology, The Affiliated Hospital, Southwest Medical University, Luzhou, China

**Keywords:** gut microbiota, gut-ovary axis, microbial metabolites, microbiome-targeted therapies, polycystic ovary syndrome

## Abstract

(PCOS), one of the most common endocrine and metabolic disorders in women of reproductive age, has a complex pathogenesis that continues to be unraveled by ongoing research. The condition is defined by three key features: hyperandrogenemia, ovulatory dysfunction, and insulin resistance. Recent studies have highlighted the gut microbiome and its metabolites as crucial regulators in PCOS development. Evidence suggests that gut dysbiosis and intestinal barrier dysfunction play a pivotal role in the onset and progression of PCOS. This review comprehensively examines the central role of gut microbiota in PCOS pathogenesis, including shifts in microbial communities such as bacteria, fungi, and viruses, and their impact on critical metabolites like short-chain fatty acids, bile acids, and tryptophan metabolites, which modulate host metabolism and reproductive function. Furthermore, based on mechanistic insights, the review explores targeted gut microbiota interventions, systematically evaluating clinical evidence for dietary modifications, probiotic/prebiotic supplementation and fecal microbiota transplantation. These approaches provide novel perspectives for precision medicine in PCOS treatment. The findings not only deepen our understanding of PCOS pathogenesis but also establish a strong theoretical foundation for innovative microbiome-based therapeutics.

## 1 Introduction

Polycystic ovary syndrome (PCOS) is one of the most common endocrine disorders affecting women of reproductive age, with a global prevalence ranging from 11% to 13% based on the Rotterdam diagnostic criteria (1, 2). For a definitive diagnosis, at least two out of three key criteria must be met: (1) oligo-ovulation or anovulation, (2) clinical or biochemical hyperandrogenism, and (3) polycystic ovarian morphology on ultrasound, after excluding other potential causes such as congenital adrenal hyperplasia or androgen-secreting tumors (2, 3). The core pathological mechanisms of PCOS involve ovulatory dysfunction, hyperandrogenism, insulin resistance (IR), and compensatory hyperinsulinemia, often accompanied by a low-grade chronic inflammatory state (3). Insulin resistance exacerbates hyperandrogenism by stimulating ovarian androgen production and reducing hepatic sex hormone-binding globulin (SHBG) synthesis, further amplifying hormonal imbalance (4). Concurrently, chronic inflammation contributes to metabolic dysregulation, creating a vicious cycle that worsens PCOS manifestations (5). These metabolic and endocrine disturbances significantly increase the risk of type 2 diabetes, cardiovascular disease, and infertility in affected women (4). Early diagnosis and targeted interventions, addressing both reproductive and metabolic aspects, are crucial in mitigating long-term complications (6).

Growing evidence suggests that the gut microbiome plays a fundamental role in maintaining host metabolic, immune, and endocrine homeostasis (7). Disruptions in microbial composition and function, commonly referred to as gut microbiome dysbiosis, have been implicated in a wide range of metabolic and inflammatory disorders, including obesity and type 2 diabetes (8). These findings highlight the broader physiological significance of host–microbiome interactions and provide a conceptual framework for exploring the contribution of gut microbial imbalance to complex endocrine disorders (9). Despite being a major global health concern, the clinical management of PCOS remains challenging due to its complex, multifactorial etiology. Genetic predisposition, environmental factors (e.g., diet, stress), insulin resistance, and intestinal barrier dysfunction all contribute to its pathogenesis (3). Moreover, current therapeutic strategies often yield suboptimal outcomes for a subset of patients, highlighting the need for novel approaches. In recent years, the gut microbiome has emerged as a key regulator of metabolic and endocrine homeostasis, offering new insights into PCOS pathophysiology (10, 11). The human gut microbiome, a diverse ecosystem of bacteria, fungi, archaea, and viruses, plays a crucial role in maintaining metabolic balance, hormonal regulation, and immune function (12, 13). Growing evidence suggests that even subtle alterations in gut microbial composition can influence systemic physiology through “gut-organ axes” (14). Notably, gut dysbiosis has been strongly linked to metabolic disorders such as obesity and type 2 diabetes, while the recently proposed gut-ovary axis offers a conceptual framework for understanding the interplay between metabolic dysfunction and reproductive abnormalities in PCOS (15). This paradigm shift underscores the potential of microbiome-targeted therapies as a novel avenue for PCOS treatment.

Building upon these pathophysiological insights, recent clinical investigations have identified distinct gut microbial signatures in PCOS patients, characterized by reduced α-diversity and specific taxonomic alterations that correlate with key clinical features (16, 17). Particularly compelling are the observed associations between certain microbial profiles and the severity of hyperandrogenism and insulin resistance - the two hallmark pathological features previously discussed (18, 19). The causal role of gut microbiota in PCOS pathogenesis has been further substantiated by animal studies demonstrating that fecal microbiota transplantation from PCOS patients to healthy mice recapitulates core disease manifestations, including anovulation and metabolic dysfunction (20, 21). These findings position the gut microbiome not merely as a bystander but as a potential active contributor to the vicious cycle of endocrine-metabolic disturbances described earlier. However, critical questions remain regarding whether microbial dysbiosis acts as a primary driver or secondary amplifier in PCOS development - a distinction that carries important therapeutic implications (22, 23). In light of these emerging findings, this review systematically evaluates current evidence on gut microbiota’s mechanistic involvement in PCOS, with particular attention to microbial metabolites and their systemic effects, while critically assessing the translational potential of microbiome-targeted interventions for this complex endocrine disorder.

## 2 The gut-ovary axis

**Figure 1:**
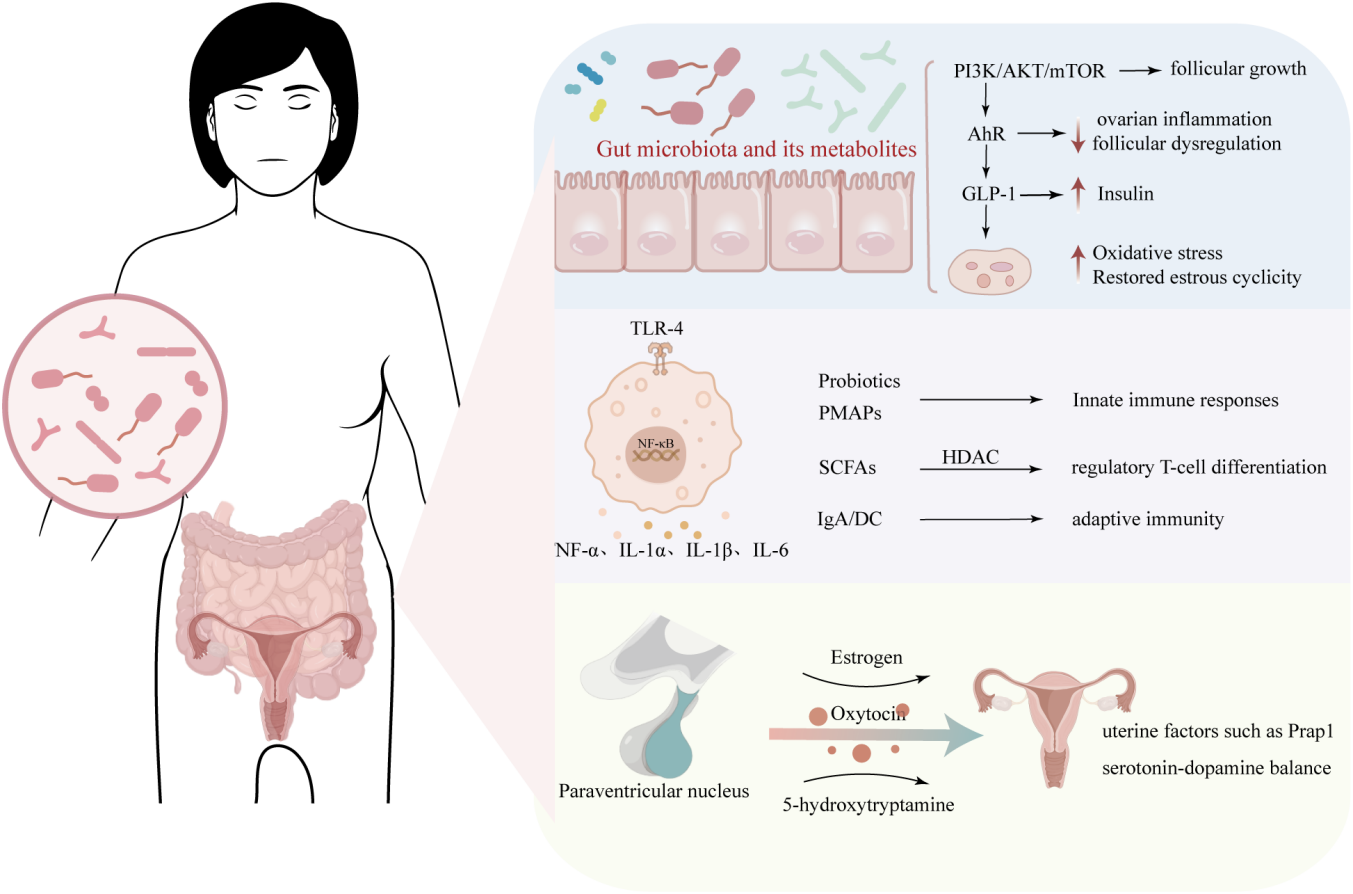
Strategies for modulating the gut-ovary axis through microbiota-targeted interventions. The gut microbiota and ovaries maintain bidirectional communication through integrated metabolic, immunologic, and neuroendocrine pathways. Microbial metabolites influence follicular development, ovulation, and insulin sensitivity via PI3K/AKT/mTOR, AhR, and GLP-1 signaling. Immunologic regulation involves probiotics, SCFAs, IgA, and Akkermansia in modulating T-cell differentiation, cytokine release, and epithelial barrier function. Neuroendocrine interactions include microbial regulation of serotonin and oxytocin pathways, which affect estrogen signaling, uterine factors, and neurotransmitter balance. Together, these networks sustain female reproductive homeostasis.

The gut microbiota and ovaries engage in sophisticated bidirectional crosstalk through an integrated network of metabolic, immunologic, and neuroendocrine pathways. These systems operate synergistically rather than in isolation, collectively maintaining female reproductive homeostasis through three principal mechanisms (**Figure 1****).**

### 2.1 Metabolic regulation via microbial metabolites

The gut microbiota exerts profound metabolic control over ovarian function through an intricate network of microbial-derived signaling molecules. Beyond the established roles of short-chain fatty acids (SCFAs) and bile acids, recent findings reveal that branched-chain amino acids, trimethylamine N-oxide, tryptophan and indole derivatives, have been implicated in the pathogenesis of metabolic disorders (24, 25). Certain Clostridium species produce indole metabolites that activate pancreatic glucagon-like peptide-1(GLP-1) secretion, enhancing glucose-stimulated insulin release (25). Beyond GLP-1–mediated effects, the gut-derived indole metabolite indole-3-propionic acid (IPA) has been shown to ameliorate PCOS in mice by restoring estrous cyclicity, improving insulin sensitivity, and reducing ovarian oxidative stress by activating the aryl hydrocarbon receptor (AhR) (26). Additionally, gut-derived urolithin A, a polyphenol metabolite, has been demonstrated to enhance oocyte quality and extend reproductive lifespan. These beneficial effects are primarily attributed to its antioxidant properties and ability to activate autophagy (27, 28). Moreover, Urolithin A suppresses primordial follicle activation via downregulation of phosphoinositide 3-kinase/Ak strain transforming (PI3K/Akt) signaling reactivation (29). These findings highlight an elaborate gut-ovary endocrine axis where microbial metabolites function as critical metabolic coordinators, integrating systemic energy status with local ovarian requirements for optimal reproductive function.

### 2.2 Immunomodulatory coordination

The gut microbiota plays a crucial role in maintaining immune homeostasis. Microbiota-derived metabolites are key mediators of host-microbe interactions (30). Some are beneficial for host physiology, such as SCFAs and secondary bile acids. Microbial-derived SCFAs (butyrate, acetate, propionate) not only enhance metabolic functions but also promote regulatory T-cell differentiation via histone deacetylase inhibition, creating an anti-inflammatory milieu. Furthermore, tryptophan catabolites shape immune responses, for instance by binding to the AhR. The AhR is highly expressed at mucosal surfaces and, when activated, enhances intestinal epithelial barrier function and regulatory immune responses (30). The microbiota also influences adaptive immunity by regulating IgA secretion and dendritic cell maturation (31). Importantly, bacterial extracellular vesicles carry immunomodulatory molecules that mediate cross-talk between gut microbes and distant organs, including ovaries (32). This multidimensional immunoregulation maintains homeostasis while preventing both excessive inflammation and immune tolerance. Emerging evidence suggests specific taxa (e.g., Akkermansia) may upregulate tight junction proteins, reducing systemic endotoxin translocation and subsequent Toll-like receptor (TLR) activation (33). Moreover, secreted Akkermansia muciniphila threonyl-tRNA synthetase functions to monitor and modulate immune homeostasis (34). Such mechanisms collectively preserve ovarian function by balancing inflammatory signals and creating a favorable follicular microenvironment.

### 2.3 Bidirectional neuroendocrine crosstalk

In addition to regulating host metabolism and immune responses, the gut microbiota is recognized to play a critical role in the production of neurotransmitters and engages in complex bidirectional communication with the hypothalamic–pituitary–gonadal (HPG) axis, contributing significantly to the pathogenesis and potential treatment of PCOS. Research has found that a reduction and imbalance in the diversity of gut microbiota can lead to a decrease in β-glucuronidase activity, thereby reducing the conversion of estrogen and phytoestrogen into their active and circulating forms. The reduction in circulating estrogen can affect the activation of estrogen receptors, potentially leading to low-estrogen diseases such as metabolic syndrome, cardiovascular diseases, and obesity.

The study by Liang et al. (2021) is the first to report an increase in γ-aminobutyric acid (GABA)-producing species in PCOS, including *Bacteroides thetaiotaomicron*, *Bacteroides fragilis*, and *Escherichia coli*, which showed a significant positive correlation with serum luteinizing hormone (LH) levels and the LH: follicle-stimulating hormone (FSH) ratio (35). Additionally, supplementation of SCFAs to a high-fat diet (HFD) significantly reduced the release of gonadotropin-releasing hormone (GnRH) from the hypothalamus and delayed the development of the gonadal axis via the Kisspeptin-G protein-coupled receptor 54-protein kinase C-extracellular signal-regulated kinases 1/2 (Kiss1-GPR54-PKC-ERK1/2) pathway, thereby reversing obesity-induced precocious puberty in female rats (36). Conversely, the brain communicates with the gut to regulate key gastrointestinal functions, including motility, permeability, pH, mucus production, immune response, and gut microbiota composition (37). The overall composition of the gut microbiota is influenced by PCOS pathology. Hyperandrogenism in PCOS alters the gut microbiota composition by promoting mucus-degrading bacteria (such as *Ruminococcus*), while depleting butyrate-producing bacteria (like *Faecalibacterium*), thereby exacerbating intestinal barrier dysfunction (38). The “estrobolome” extends beyond estrogen reactivation to include microbial modulation of androgen metabolism - specific Bacteroides strains express hydroxysteroid dehydrogenases that convert androgens to less active forms (39). This microbial-endocrine crosstalk creates either vicious or virtuous cycles: PCOS-associated dysbiosis worsens hormonal imbalance.

## 3 Role of intestinal barrier dysfunction in the pathogenesis of PCOS

**Figure 2:**
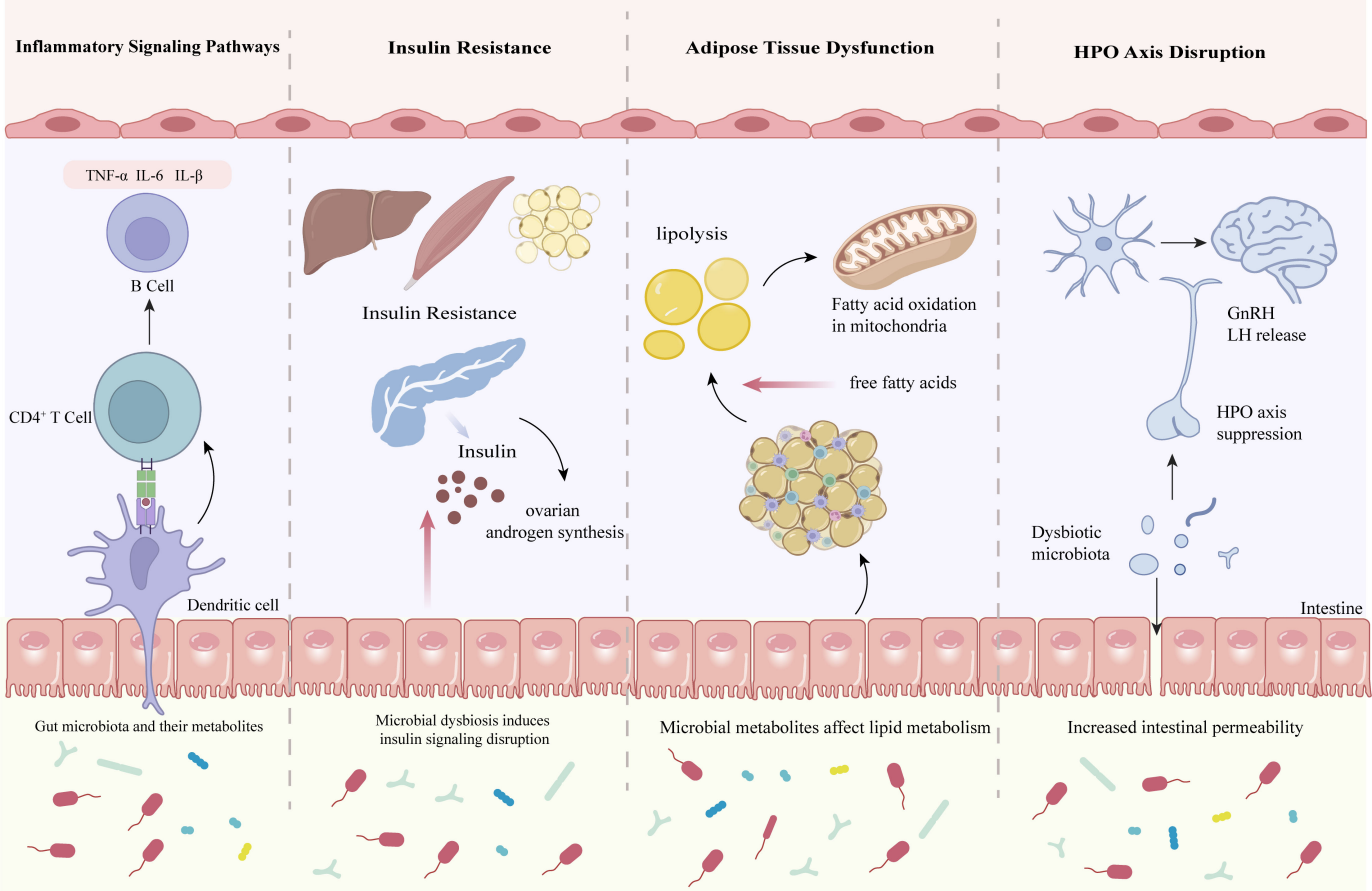
Role of intestinal barrier dysfunction in the pathogenesis of PCOS. Disruption of the intestinal barrier permits bacterial components such as lipopolysaccharide (LPS) to enter the circulation, initiating immune dysregulation and metabolic disturbances. (Left) Inflammatory signaling: LPS activates dendritic cells and CD4^+^ T cells, leading to NF-κB–mediated cytokine release and B cell activation. (Middle-left) Insulin resistance: systemic inflammation and dysbiosis exacerbate insulin resistance in liver, muscle, and adipose tissue, while hyperinsulinemia promotes ovarian androgen synthesis. (Middle-right) Adipose tissue dysfunction: increased lipolysis elevates circulating free fatty acids, further aggravating metabolic imbalance. (Right) HPO axis disruption: altered gut-derived signals and vagal activation impair hypothalamic–pituitary–ovarian communication. Collectively, intestinal barrier dysfunction links gut dysbiosis to immune, metabolic, and neuroendocrine alterations in PCOS.

The intestinal barrier, composed of the mucus layer, epithelial cells with their tight junction proteins (e.g., Zonula Occludens-1 (ZO-1), occludin), and immune cells, serves as a critical structure for maintaining gut homeostasis and preventing the translocation of pathogens and toxins (40). When its integrity is compromised, it can lead to abnormally increased intestinal permeability (termed “leaky gut”), allowing gut microbiota and microbial products to translocate from the intestinal lumen into systemic circulation (41, 42). Bacterial cell wall components, such as lipopolysaccharide (LPS, endotoxin), enter the bloodstream, triggering immune dysregulation and metabolic disturbances (**Figure 2**).

### 3.1 Activation of inflammatory signaling pathways

Research indicates that increased intestinal permeability allows endotoxins such as LPS to enter the systemic circulation, where they trigger systemic inflammatory responses by activating the Toll-like receptor 4/nuclear factor kappa-light-chain-enhancer of activated B cells (TLR4/NF-κB) inflammatory pathway. Specifically, circulating LPS binds to TLR4 on immune cell surfaces, activating the downstream NF-κB signaling pathway and promoting the release of pro-inflammatory cytokines such as tumor necrosis factor-alpha (TNF-α), interleukin-6 (IL-6), and interleukin-1 beta (IL-1β). This process establishes a chronic low-grade inflammatory state. Direct evidence for this mechanism comes from a study by Hu et al. (2021), which demonstrated significant activation of the TLR4/NF-κB pathway in the endometrial tissue of PCOS patients (43). This persistent inflammatory microenvironment exerts multifaceted effects on PCOS pathogenesis: on one hand, it disrupts normal follicular development, promotes ovarian fibrosis and granulosa cell apoptosis, and exacerbates ovulatory dysfunction and the formation of polycystic ovarian morphology; on the other hand, it aggravates insulin resistance by interfering with insulin signaling, while simultaneously stimulating androgen synthesis in the ovaries and adrenal glands. This cycle drives the onset and progression of PCOS, highlighting the critical role of gut-derived inflammation in the disease’s pathophysiology.

### 3.2 Exacerbation of insulin resistance

Studies confirm that LPS and its induced inflammatory cytokines (e.g., TNF-α) directly disrupt insulin signaling. Hotamisligil et al. (1996) first demonstrated that TNF-α induces insulin resistance by promoting serine phosphorylation of insulin receptor substrate 1(IRS-1), thereby inhibiting the tyrosine kinase activity of the insulin receptor (44). Subsequent studies further revealed that both TNF-α and LPS activate the c-Jun N-terminal kinase (JNK) pathway to phosphorylate IRS-1 at Ser307, which blocks PI3K/Akt signaling and consequently impairs glucose uptake (45). This process additionally involves the participation of oxidative stress—LPS suppresses the nuclear factor erythroid 2-related factor 2 (Nrf2) antioxidant pathway, upregulates NADPH oxidase 2/4(NOX2/4) and inducible nitric oxide synthase (iNOS) expression in adipocytes, increases reactive oxygen species (ROS) production, and reduces the activity of antioxidant enzymes such as manganese superoxide dismutase (MnSOD) and heme oxygenase-1(HO-1), further compromising IRS expression and GLUT4 function (46). Persistent insulin resistance triggers hyperinsulinemia, which in turn promotes hyperandrogenemia through several pathways. Concurrently, hyperinsulinemia synergizes with LH to stimulate ovarian theca cells, enhancing cytochrome P450 family 17 subfamily A member 1 (human gene CYP17A1) and 3β-hydroxysteroid dehydrogenase (3β-HSD) activity to boost androstenedione and testosterone synthesis (47). Systematic investigations by Diamanti-Kandarakis and Dunaif reveal that in PCOS patients, insulin resistance, hyperinsulinemia, and hyperandrogenemia mutually reinforce one another, creating a self-perpetuating pathological loop that is notoriously difficult to break (47).

### 3.3 The positive feedback loop of hyperandrogenemia

Studies have demonstrated that LPS can not only indirectly contribute to hyperandrogenemia by inducing insulin resistance (47), but also directly promote androgen synthesis through activation of ovarian TLR4 receptors. Experiments by Fox et al.(2021) confirmed that LPS treatment significantly increased androstenedione (a testosterone precursor) secretion in rat theca interstitial cells (TICs), accompanied by upregulation of key androgen biosynthesis genes including cytochrome P450 family 17 subfamily A member 1 (rat gene Cyp17a1) and cytochrome P450 family 11 subfamily A member 1 (rat gene Cyp11a1) (48). Furthermore, RNA sequencing analysis revealed that LPS induces comprehensive alterations in androgen synthesis pathways by regulating cholesterol metabolism (e.g., 3-hydroxy-3-methylglutaryl-CoA reductase (rat gene Hmgcr)) and inflammation-related genes. These findings provide mechanistic evidence for LPS directly stimulating ovarian local androgen excess, suggesting that chronic low-grade inflammation may be an important contributor to hyperandrogenemia in PCOS (49). Further studies revealed that hyperandrogenemia may establish a self-amplifying mechanism by upregulating the expression of steroidogenic enzymes. Using an insulin and human chorionic gonadotropin (hCG)-induced PCOS rat model, Li et al. (2013) observed a marked increase in the expression of cholesterol side-chain cleavage enzyme and 17α-hydroxylase/17, 20-lyase in ovarian theca and stromal cells, accompanied by elevated serum androgen levels (50). These findings suggest that a hyperandrogenic state may further potentiate androgen synthesis by upregulating ovarian steroidogenic enzyme expression, thereby creating a positive feedback loop.

### 3.4 Adipose tissue dysfunction and metabolic derangements

The translocation of LPS into circulation due to intestinal barrier dysfunction disrupts adipose metabolic homeostasis through multiple mechanisms. Studies demonstrate that LPS activates adipose tissue macrophages via TLR4 receptors, promoting the release of proinflammatory cytokines such as TNF-α, which in turn induces excessive lipolysis in adipocytes, generating large quantities of free fatty acids (FFAs) (51). These FFAs not only feed back to further activate macrophages, establishing a vicious cycle of “inflammation-lipolysis”, but also directly interfere with insulin signal transduction. Through lipid metabolomics and proteomics analyses, Qian et al. observed a significant increase in serum FFAs, particularly palmitic acid (C16:0) and oleic acid (C18:1), in patients with PCOS (52). These abnormally elevated FFAs may not only induce lipotoxicity but also stimulate local ovarian androgen synthesis, thereby exacerbating core PCOS symptoms. Meanwhile, studies have found that LPS can upregulate suppressor of cytokine signaling 3(SOCS3) expression through TLR4- myeloid differentiation primary response 88(MyD88) activation (53), which may indirectly interfere with the leptin signaling pathway via SOCS3 (54, 55). These changes collectively lead to adipose tissue dysfunction, characterized by increased release of inflammatory cytokines, enhanced lipolysis, impaired insulin signaling, and the development of leptin resistance, ultimately leading to the typical metabolic abnormal phenotype characteristic of PCOS (51, 53–55).

### 3.5 Hypothalamic-pituitary-ovarian axis disruption

Disruption of gut microbiota leading to systemic LPS translocation not only impairs peripheral organ function but also exacerbates PCOS pathology by interfering with the neuroendocrine regulation of the HPO axis (56). In an acyclic ewe model with intravenous LPS administration, Herman and Tomaszewska-Zaremba (2010) demonstrated that LPS treatment significantly suppressed GnRH and its receptor messenger RNA (mRNA) expression in the hypothalamic preoptic area and median eminence (by 40–60% and 50%, respectively; p ≤ 0.01), concurrently inhibiting gonadotropin-releasing hormone receptor (GnRHR) gene expression in the anterior pituitary (by 80%, p ≤ 0.01) and reducing plasma LH concentrations (by 25%, p ≤ 0.05). These findings confirm that LPS directly dampens GnRH neuronal activity via central inflammatory responses (56).

## 4 Gut microbiota characteristics in PCOS

**Figure 3:**
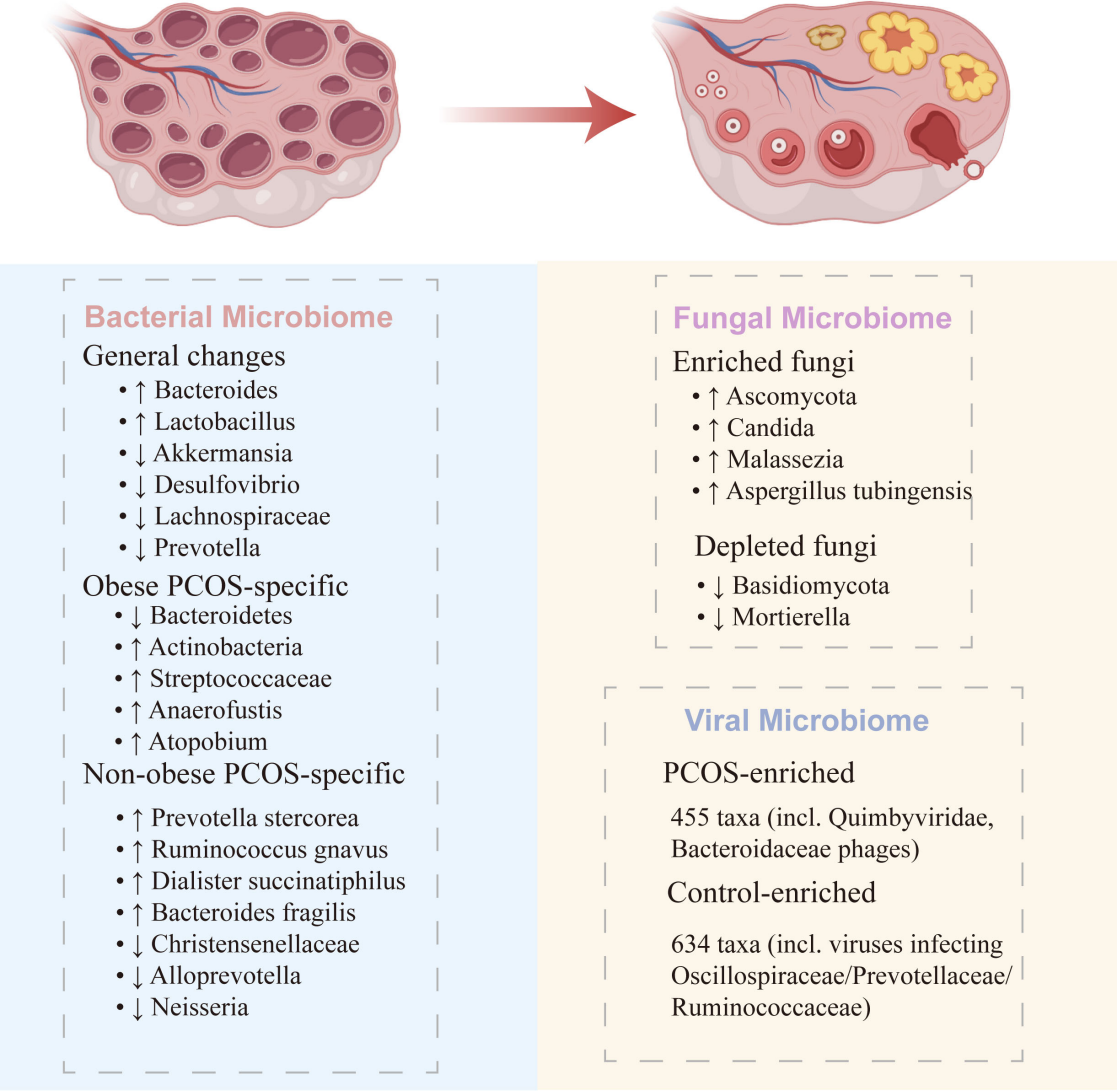
Gut microbiota characteristics in PCOS. PCOS is associated with distinct alterations in the bacterial, fungal, and viral microbiomes. Bacterial microbiome: common changes include increased *Bacteroides* and *Lactobacillus*, with decreased *Akkermansia*, *Desulfovibrio*, *Lachnospiraceae*, and *Prevotella*. Obese PCOS patients show reduced *Bacteroidetes* and elevated *Actinobacteria*, *Streptococcaceae*, and *Anaerofustis*, whereas non-obese PCOS patients exhibit enrichment of *Prevotella stercorea*, *Ruminococcus gnavus*, and *Dialister succinatiphilus*, alongside depletion of *Alloprevotella* and *Neisseria*. Fungal microbiome: increased *Ascomycota*, *Candida*, *Malassezia*, and *Aspergillus tubingensis*, with reductions in *Basidiomycota* and *Mortierella*. Viral microbiome: PCOS patients harbor 455 enriched taxa (e.g., *Quimbyviridae*, *Bacteroidaceae* phages), while controls show enrichment of 634 taxa, including viruses infecting *Oscillospiraceae*, *Prevotellaceae*, and *Ruminococcaceae*. These findings provide experimental evidence linking gut microbial dysbiosis to PCOS pathophysiology.

Recent studies have shown distinct differences in gut microbial composition between PCOS and healthy controls. Chen et al. (2023) conducted 16S rRNA and internal transcribed spacer (ITS) sequencing analyses comparing 17 PCOS patients with 17 matched controls, revealing significant alterations in microbial composition and functional profiles in the PCOS group (17). Clinical studies by Lindheim et al. (16), Torres et al. (18), and Liu et al. (57) similarly confirmed the presence of gut microbiota dysbiosis in PCOS patients, while animal experiments conducted by Kelley et al. (58) and Guo et al. (59) provided further experimental evidence supporting these findings (**Figure 3**; **Table 1**).

**Table 1:** Studies of gut microbiome alterations in PCOS.

Study	Year	Population/model	Microbiome domain	Overall characteristics in PCOS	Representative alterations	Key findings
Torres et al. (18)	2018	PCOS n=73; PCOM n=42; controls n=48	Bacterial microbiome	α-diversity ↓; Faith’s PD ↓; β-diversity altered	*Porphyromonas* spp. ↑; *B. coprophilus* ↑; *Blautia* spp. ↑; *F. prausnitzii* ↑; *Anaerococcus* spp. ↓; *Odoribacter* spp. ↓; *Roseburia* spp. ↓; *R. bromii* ↓	lower diversity and microbial shifts
Zeng et al. (19)	2018	HC n=8; NIR-PCOS n=8; IR-PCOS n=9	Bacterial microbiome	α-diversity ↓; β-diversity altered; dysbiosis more pronounced in IR-PCOS; steroid hormone biosynthesis ↑; LPS biosynthesis ↑	Bacteroidaceae ↑; *Bacteroides* ↑; Prevotellaceae ↓; *Prevotella 9* ↓; *Faecalibacterium* ↓; Lachnospiraceae ↓; Ruminococcaceae ↓	Gut dysbiosis was most evident in IR-PCOS; IR status should be considered in PCOS microbiome stratification
Jobira et al. (64)	2020	Obese adolescents: PCOS n=37; controls n=21	Bacterial microbiome	α-diversity ↓; β-diversity altered	Actinobacteria ↑; Bacteroidetes ↓; Bacteroidaceae ↓; Porphyromonadaceae ↓; Streptococcaceae ↑; *Prevotella* ↑; *Finegoldia* ↑; *Lactobacillus* ↑; *Bacteroides* ↓; *Parabacteroides* ↓	Dysbiosis was evident in obese adolescent PCOS: altered taxa; testosterone and metabolic abnormalities
Dong et al. (65)	2021	PCOS n=45; controls n=37	Bacterial microbiome	α-diversity ↓ (trend); dysbiosis; LPS biosynthesis-related pathways ↑	*R. gnavus* ↑; *P. stercorea* ↑; *D. succinatiphilus* ↑; *B. fragilis* ↑; *Christensenellaceae* spp. ↓	*P. stercorea* ↑ in non-IR/non-overweight PCOS; gut microbiota alterations; endocrine and metabolic disturbances
Yin et al. (68)	2022	Healthy-LB n=21; PCOS-LB n=22; Healthy-HB n=20; PCOS-HB n=25	Bacterial and fungal microbiome	Bacterial/fungal diversity altered; community structure changed; BMI-stratified dysbiosis	Bacteria: R. torques ↑; Escherichia/Shigella ↑; Lachnoclostridium ↑; Fungi: Candida ↑; Malassezia ↑; Mortierella ↓	Distinct bacterial and fungal dysbiosis in PCOS; BMI and hyperandrogenism may shape microbial patterns
Wu et al. (69)	2025	PCOS n=118; controls n=108; mouse models	Fungal microbiome	Gut mycobiota dysbiosis; fungal metabolite changes observed	*Aspergillus tubingensis* ↑; *Candida tropicalis* ↑;	*A. tubingensis* and its metabolite AT-C1: PCOS pathogenesis; causal fungal contribution supported in mice
Huang et al. Huang. (70)	2022	PCOS n=50; controls n=43	Viral microbiome	Viral α-diversity ↓; virome composition altered	Siphoviridae ↓; Quimbyviridae ↑; Bacteroidaceae phages ↑; control-enriched viruses infecting Oscillospiraceae/Prevotellaceae/Ruminococcaceae ↑	Gut virome dysbiosis in PCOS; altered virus-bacteria interactions; diagnostic potential ↑ ↑ indicates increase/upregulation and ↓ indicates decrease/downregulation.

↑ indicates increase/upregulation and ↓ indicates decrease/downregulation.

### 4.1 Bacterial microbiome

Emerging evidence from both human and rodent studies suggests that gut microbial dysbiosis may be a potential pathogenic factor in PCOS development (60). Multiple studies have confirmed a significant reduction in gut bacterial microbiome diversity among patients with PCOS. Torres et al.(2018) analyzed the gut microbiota of 73 PCOS patients and 48 healthy controls, revealing markedly decreased Observed species and Faith phylogenetic diversity (PD) in the PCOS group, which were inversely correlated with serum testosterone levels (18). These findings were further validated in a meta-analysis of 28 studies (involving 1,022 PCOS patients and 928 controls), which demonstrated significantly lower α-diversity in PCOS patients (Shannon index: SMD = -0.27; *P* < 0.05), alongside depletion of beneficial bacteria such as *Lachnospira* and *Prevotella* and enrichment of pro-inflammatory genera like *Bacteroides* and *Lactobacillus (*61). Recent studies also identified a notable decline in the abundance of *Akkermansia* and *Desulfovibrio* in PCOS patients (62). As a key commensal bacterium for maintaining gut barrier integrity, the reduction of *Akkermansia* may exacerbate hyperandrogenemia and ovulatory dysfunction in PCOS by increasing LPS translocation, triggering ovarian macrophage pyroptosis and interferon-γ (IFN-γ)-mediated inflammatory responses, thereby disrupting steroid hormone synthesis, promoting granulosa cell apoptosis, and exacerbating hyperandrogenemia and ovulatory dysfunction in PCOS. Notably, alterations in gut microbiota are closely linked to body mass index (BMI). Research indicates that, regardless of sex or PCOS status, BMI correlates positively with the abundance of genera such as *Anaerofustis* and *Atopobium* but negatively with *Alloprevotella* and *Neisseria (*63). Furthermore, fat mass exhibits even stronger associations with these bacterial genera, while fasting glucose levels correlate positively with *Raoultella* abundance, and insulin sensitivity indices correlate negatively with *Atopobium* and *Scardovia*. This BMI-dependent microbial pattern suggests that PCOS patients with varying BMI statuses may harbor distinct gut microbiota profiles.

In obese PCOS patients, gut microbiota alterations are more pronounced. Jobira et al.(2020) conducted a prospective case-control study demonstrating that obese adolescent PCOS patients exhibit reduced α-diversity (Shannon diversity), significantly lower relative abundance of *Bacteroidetes*, and increased *Actinobacteria* and *Streptococcaceae*, all of which closely correlate with testosterone levels and insulin resistance (64). Similarly, Zeng et al.(2019) observed that in insulin-resistant PCOS (IR-PCOS) patients, enrichment of *Bacteroidaceae* and depletion of *Prevotellaceae* were significantly associated with metabolic parameters. Additionally, marked differences in *Ruminococcaceae* and *Lachnospiraceae* were noted. Phylogenetic investigation of communities by reconstruction of unobserved states functional prediction revealed alterations in 73 metabolic pathways, including enhanced glycolysis and steroid hormone biosynthesis, clearly indicating a microbial functional shift toward energy harvest (19). These findings collectively suggest that the gut microbiota characteristics of obese PCOS patients may exacerbate metabolic abnormalities through multiple mechanisms, including disrupted energy metabolism, promotion of low-grade inflammation, and impaired gut barrier function.

In contrast, non-obese PCOS patients exhibit unique gut microbiota patterns. Dong et al. (2021) employed full-length 16S rDNA sequencing to analyze 45 PCOS patients and 37 healthy women, revealing that *Prevotella stercorea* was significantly enriched in the non-obese PCOS (NOW) group compared to non-obese controls (*P* < 0.05), with similar enrichment observed in the non-insulin-resistant subgroup (65). Additionally, non-obese PCOS patients showed increased abundance of *Ruminococcus gnavus*, *Dialister succinatiphilus*, and *Bacteroides fragilis*, alongside depletion of *Christensenellaceae* spp. Notably, Kyoto Encyclopedia of Genes and Genomes (KEGG) analysis highlighted significant enrichment of LPS biosynthesis pathways in the PCOS group, suggesting that gut microbiota may contribute to PCOS pathogenesis by modulating inflammatory responses (65). These findings provide novel insights into the pathophysiology of non-obese PCOS patients, particularly the potential interplay between gut microbiota and metabolic dysregulation.

### 4.2 Fungal microbiome

The role of the gut fungal microbiome (mycobiome) in health, disease, and clinical applications has gained increasing attention in recent years (66). Although fungi represent a small fraction (0.01%–0.1%) of the microbial community, their larger size and distinct immunoregulatory roles underscore their importance. Factors such as diet, antimicrobial use, and age can disrupt the fungal microbiota, resulting in dysbiosis (67). Clinical studies have revealed that PCOS patients exhibit significantly reduced gut fungal diversity and altered community structure, characterized by an increase in Ascomycota and a decrease in Basidiomycota, alongside notable enrichment of pathogenic fungi such as Candida and Malassezia (17). Further analysis using 16S rRNA and ITS2 gene sequencing by Yin et al.(2022) demonstrated that normal-weight PCOS patients had the lowest fungal diversity, with distinct compositional differences compared to healthy individuals. Specifically, commensal fungi like Mortierella were more abundant in healthy controls, suggesting potential metabolic protective effects (68). These findings highlight a possible critical link between gut fungal dysbiosis and PCOS pathogenesis.

Emerging research has further uncovered direct pathogenic roles of specific fungi in PCOS. Wu et al.(2025) employed ITS2 sequencing and culture-dependent strategies to identify significant enrichment of Aspergillus tubingensis in the gut of PCOS patients. This fungus secretes a secondary metabolite, AT-C1, which suppresses the AhR signaling pathway, reducing interleukin-22(IL-22) secretion by group 3 innate lymphoid cells (ILC3) in the gut, ultimately inducing PCOS-like phenotypes in mouse models (69). This discovery not only establishes a causal role for gut fungi in PCOS but also provides a theoretical foundation for therapies targeting fungi and their metabolites. However, no studies to date have precisely delineated the mechanistic interactions between “specific fungi and bacteria” in PCOS pathogenesis (17), and whether these microbial alterations are a cause or consequence of PCOS remains to be validated with higher-level evidence.

### 4.3 Viral microbiome

Beyond bacteria and fungi, the gut virome—an essential component of the gut ecosystem—has also been implicated in disease development through its regulation of bacterial communities and host immunity (60). Huang et al.(2022) conducted the first systematic characterization of gut virome alterations in PCOS using metagenomic sequencing of fecal samples from 50 patients and 43 healthy women (70). Their study revealed significantly reduced viral α-diversity and distinct shifts in viral community structure in PCOS patients, marked by decreased Siphoviridae and enriched Quimbyviridae.Viral operational taxonomic unit (vOTU) analysis identified 1,089 differentially abundant viral taxa, with 455 enriched in PCOS (including multiple Bacteroidaceae phages) and 634 enriched in controls (primarily viruses infecting Oscillospiraceae, Prevotellaceae, and Ruminococcaceae) (70). Notably, a virome-based disease prediction model demonstrated exceptional diagnostic accuracy (area under the curve (AUC) = 0.938), suggesting the gut virome as a novel biomarker for PCOS. These findings not only bridge a gap in PCOS virome research but also offer critical insights into virus-bacteria interactions in PCOS pathogenesis.

Nevertheless, current studies on PCOS and the gut microbiome, particularly the fungal and viral components, remain limited, often constrained by small sample sizes, inadequate control for confounders, lack of causal mechanistic validation, and insufficient stratification of PCOS subtypes.

## 5 Microbial metabolites in PCOS pathogenesis

Alterations in gut microbiome diversity and composition represent one of the key features of dysbiosis observed in PCOS (71, 72). Beyond taxonomic shifts, these microbial changes lead to functional alterations in microbial metabolic activity, resulting in modified production of bioactive metabolites that can influence multiple host physiological systems, including the gut–ovary axis (73, 74). Microbial metabolites play a pivotal role in the onset and progression of PCOS by modulating the host’s metabolic milieu. Gut microbes produce or metabolize a spectrum of bioactive compounds, including SCFAs, bile acids, and tryptophan derivatives, which collectively influence energy homeostasis, insulin sensitivity, and sex hormone balance. Dysbiosis-driven aberrations in microbial composition and activity disrupt the production of these metabolites, compromising intestinal barrier integrity, fueling chronic low-grade inflammation, and exacerbating insulin resistance. These perturbations ultimately promote theca cell hyperplasia and ovulatory dysfunction. By elucidating the molecular interactions between microbial metabolites and host cells, we can not only uncover novel insights into the metabolic origins of PCOS but also establish a theoretical foundation for precision intervention strategies targeting microbial metabolic reprogramming.

### 5.1 Short-chain fatty acids

SCFAs, including acetate, propionate, and butyrate, are key microbial metabolites produced through the fermentation of dietary fibers by gut microbiota. These bioactive compounds play crucial roles in maintaining metabolic homeostasis, immune regulation, and endocrine function through multiple interconnected mechanisms (75). SCFAs exert their effects primarily by activating G protein-coupled receptors, including free fatty acid receptor 2 (FFAR2/GPR43) and free fatty acid receptor 3 (FFAR3/GPR41), which enhances the secretion of gut hormones like GLP-1 and peptide YY (PYY) to improve insulin sensitivity and modulate HPG axis function. Additionally, SCFAs maintain intestinal barrier integrity by upregulating tight junction proteins, thereby preventing LPS translocation and subsequent systemic inflammation. However, SCFA metabolism is significantly disrupted in PCOS patients. Clinical studies by Zhang et al. (2019) revealed that levels of acetate, propionate, butyrate, and valerate were markedly lower in PCOS patients compared to healthy controls (76). Notably, supplementation with the probiotic Bifidobacterium lactis V9 elevated SCFA levels in a subset of PCOS patients (9/14), correlating with successful probiotic colonization in the gut (76). Animal studies further demonstrated that sodium acetate intervention (200 mg/kg) improved ovarian function in PCOS model rats by suppressing histone deacetylase (HDAC) activity, evidenced by normalized follicular growth and elevated circulating 17-β estradiol levels (77).

At the mechanistic level, dysregulation of SCFAs contributes to the pathogenesis of PCOS through multiple pathways: first, decreased SCFAs (including butyrate) reduce the expression of intestinal tight junction proteins, increasing gut permeability and allowing LPS translocation into circulation, which triggers chronic low-grade inflammation; second, SCFAs enhance insulin signaling in adipose tissue and liver via FFAR2/FFAR3 (GPR43/GPR41) receptor activation, and their deficiency directly exacerbates insulin resistance; third, SCFAs modulate the secretion of GLP-1 and PYY, indirectly influencing HPG axis function, with their dysregulation linked to hyperandrogenism and ovulatory dysfunction; furthermore, SCFA deficiency upregulates pro-inflammatory cytokines such as IL-6 and TNF-α, aggravating systemic low-grade inflammation (78). In terms of immunomodulation, butyrate enhances interleukin-10(IL-10) secretion by regulatory B cells (B10) and promotes regulatory T cells (Treg) differentiation through HDAC inhibition, while simultaneously suppressing inflammatory responses (79). This immune imbalance exacerbates ovarian inflammation and insulin resistance via elevated interleukin-17(IL-17) and TNF-α, suggesting that SCFA-mediated metabolic modulation may represent a novel therapeutic strategy for multi-system interventions in PCOS.

### 5.2 Bile acids

In recent years, mounting evidence has highlighted the pivotal role of bile acids in metabolic diseases, with a growing body of research exploring their association with PCOS. Yu et al. (2023) employed high-performance liquid chromatography-tandem mass spectrometry (LC/MS) to analyze 408 PCOS patients, revealing significantly elevated serum levels of chenodeoxycholic acid (CDCA) and cholic acid (CA), which positively correlated with insulin resistance and hyperandrogenemia. The team established a predictive model for PCOS based on a combination of five bile acids (AUC = 0.86), underscoring bile acids as early warning biomarkers for metabolic dysfunction (80). In a cross-sectional study of lean PCOS women, Zhu et al. (2024) demonstrated that CDCA exhibited a linear positive correlation with total testosterone (r = 0.130, *P* = 0.044) and free testosterone (r = 0.153, *P* = 0.019). They also identified a characteristic “deoxycholic acid (DCA) decrease-CDCA increase” pattern, suggesting this bile acid fingerprint as an independent risk factor for hyperandrogenemia in lean PCOS (81).

Aberrant bile acid metabolism contributes to the pathogenesis of PCOS through intricate molecular mechanisms. Yang Y.L. et al.(2021) found that CDCA enhanced glucose metabolism in PCOS mice (82). Additionally, studies have reported that bile acids not only influence spermatocyte apoptosis and sperm function in male mice (83) but also ameliorate ovarian ischemia-reperfusion injury (84). The Qi research team, through fecal microbiota transplantation and *Bacteroides vulgatus* colonization experiments, demonstrated that aberrant proliferation of *B. vulgatus* in the gut microbiota of PCOS patients led to markedly reduced levels of glycodeoxycholic acid (GDCA) and tauroursodeoxycholic acid (TUDCA). This alteration suppressed GATA3 expression via the bile acid-Takeda G protein-coupled receptor 5(TGR5)-cyclic adenosine monophosphate (cAMP) signaling pathway, leading to a marked ~90% reduction in IL-22 secretion from intestinal group 3 innate lymphoid cells (ILC3s), which subsequently induced insulin resistance, ovarian dysfunction (characterized by increased follicular cysts and decreased corpora lutea), and impaired fertility (20). Moreover, microbial enzymes, notably bile salt hydrolase (BSH), hydrolyze primary bile acids into secondary bile acids. This conversion allows secondary bile acids to activate receptors like the farnesoid X receptor (FXR) and the G-protein-coupled receptor TGR5. which modulates metabolism and hormonal processes, including HPO axis signaling, to promote ovarian androgen production and increase testosterone (82).

### 5.3 Tryptophan metabolites

Tryptophan metabolites are generated either through direct decomposition of tryptophan by gut microbiota (e.g., *Clostridium, Escherichia coli*) into indole derivatives (such as IPA and indole-3-acetic acid (IAA)) or via host-cell-mediated processes (e.g., indoleamine 2,3-dioxygenase 1(IDO1)-catalyzed kynurenine production, 5-hydroxytryptamine (5-HT) synthesis by enterochromaffin cells) in coordination with microbial metabolism. These metabolites regulate intestinal barrier integrity, energy metabolism, and ovarian function by activating the AhR, modulating immune responses (e.g., IL-22), and influencing neuroendocrine pathways (e.g., 5-HT signaling (85–87). In PCOS patients, this metabolic network is markedly disrupted: da Silva et al. (2024) employed 16S rRNA sequencing and liquid chromatography-triple quadrupole-mass spectrometry (LC-QqQ-MS) to analyze a cross-sectional cohort of 38 women (24 with PCOS), revealing significantly reduced serum IPA levels in the PCOS group. Multivariate analysis confirmed that low IPA levels were independently associated with PCOS (73). Li et al. (2025) further demonstrated in animal studies that IPA supplementation inhibited the NLR family pyrin domain containing 3(NLRP3) inflammasome via AhR activation, ameliorating dehydroepiandrosterone (DHEA)-induced PCOS phenotypes in mice, including restored ovarian morphology, improved insulin sensitivity, and normalized hormone levels (26). Concurrently, Wang et al. conducted a clinical study of 400 women (200 with PCOS), showing aberrant activation of the tryptophan-kynurenine pathway in PCOS patients, with significantly elevated plasma levels of tryptophan, kynurenine, and quinolinic acid. These metabolites positively correlated with LH, anti-Müllerian hormone (AMH), and insulin resistance markers, and their combined diagnostic value for PCOS yielded an AUC of 0.824 (88). Collectively, these findings underscore the multifaceted role of the gut microbiota-tryptophan metabolic axis in the pathogenesis of PCOS.

### 5.4 Other metabolites

In addition to short-chain fatty acids, bile acids, and tryptophan metabolites, numerous other gut microbial metabolites are implicated in the pathogenesis of PCOS. For instance, as previously mentioned, Wu et al. (2025) discovered that *Talaromyces purpurogenus*, enriched in the gut of PCOS patients, produces the metabolite AT-C1, which suppresses the AhR signaling pathway and reduces IL-22 secretion, thereby inducing PCOS-like symptoms (69). Similarly, gut microbiota-derived trimethylamine-N-oxide (TMAO) has been closely linked to metabolic disturbances in PCOS. Huang et al. (2025) demonstrated that in a DHEA-induced hyperandrogenemic PCOS mouse model, plasma TMAO levels were significantly reduced. TMAO supplementation restored oocyte quality and reproductive potential by ameliorating mitochondrial function and reducing oxidative stress (89). Notably, beyond directly pathogenic metabolites like TMAO, certain gut microbes can produce molecules with dual regulatory effects through metabolic reprogramming. Yun et al. (2024) recently discovered that guanidine, produced by Bacteroides vulgatus, although a microbial metabolite, has a mechanism of action that is completely different from that of TMAO. It specifically activates the FXR receptor to inhibit GLP-1 secretion, thereby exacerbating PCOS symptoms through another pathway (90). However, the roles of gut microbial metabolites are complex, and some may confer protective effects in PCOS. For instance, polyamines such as spermidine exhibit protective effects, with animal studies confirming their ability to improve follicular development by enhancing ovarian antioxidant capacity and modulating autophagy (91). Collectively, these findings highlight how gut microbiota, through their diverse metabolite networks, intricately influence PCOS progression via both disease-promoting and protective mechanisms.

## 6 Therapeutic modulation of the microbiome in PCOS

**Figure 4:**
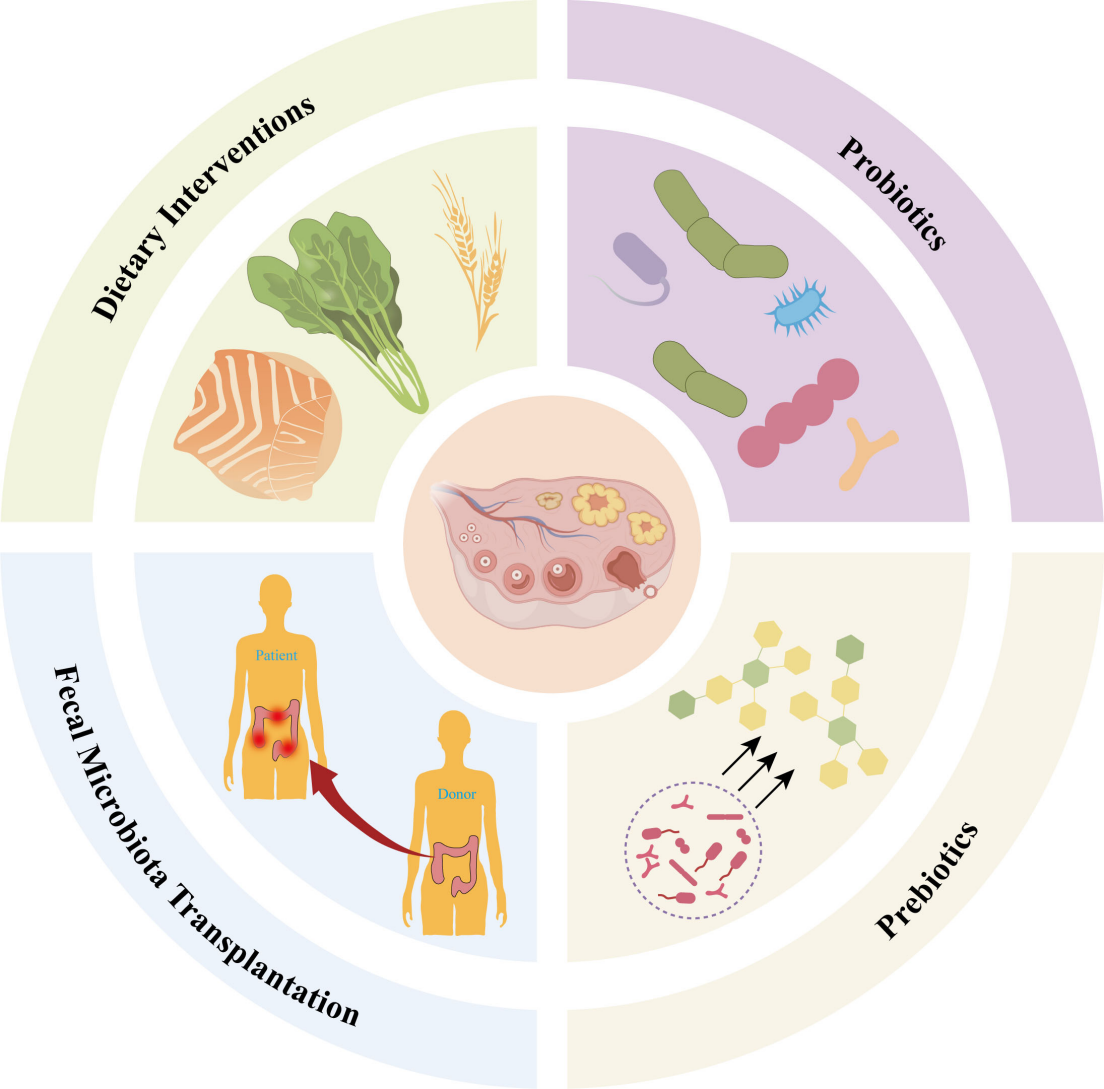
Microbiome-targeted therapeutic strategies in PCOS. The central panel illustrates ovarian physiology, surrounded by four major approaches for gut microbiota modulation: (i) Dietary interventions (e.g., whole grains, leafy vegetables, and omega-3 rich fish) to improve microbial composition and host metabolism; (ii) Probiotics, consisting of beneficial live microorganisms such as Lactobacillus and Bifidobacterium species; (iii) Prebiotics, including nondigestible dietary fibers and oligosaccharides that selectively promote the growth of beneficial gut bacteria (arrows indicate stimulation of microbial populations); and (iv) Fecal microbiota transplantation (FMT), whereby gut microbiota from a healthy donor are transferred to a patient to restore microbial balance. Collectively, these interventions act on the intestinal microbiota to regulate inflammation, metabolism, and hormone signaling, ultimately influencing ovarian follicle development and function.

In recent years, despite growing research interest in the gut microbiome, clinical trials targeting microbiome-based therapies for PCOS remain limited. Emerging evidence suggests that dietary modifications may serve as a viable strategy to ameliorate metabolic and reproductive dysfunction in PCOS (92). Concurrently, novel investigations are exploring targeted microbial therapies, including probiotics, prebiotics, and fecal microbiota transplantation (FMT), to optimize therapeutic outcomes for PCOS patients (**Figure 4**, **Table 2**).

**Table 2:** Summary of studies investigating microbiota-targeted interventions in PCOS.

Study	Year	Sample size	Effect on gut microbes	Effect on PCOS
Geng et al. (99)	2025	Mice: n=5-6/group Clinical: n=45 FMT: n=5-8/group	↑ *Bifidobacterium*, SCFAs-producers, CAG12/CAG16, CAZy genes, SCFAs ↓ F/B ratio, *Lachnospiraceae*, α-diversity	Clinical: ↓ Testosterone, DHEAs, AMH, FBG, FIN, HOMA-IR, BMI, TC Mice: ↑ GTT/ITT, BAT thermogenesis, estrous cycles, corpora lutea, gut barrier ↓ Testosterone, AMH, LH/FSH, cystic follicles, LBP, IL-1β, IL-18, ovarian pyroptosis FMT: Post-inulin microbiota recapitulated these effects
Luo et al. (107)	2023	Mice: n=30 Clinical: n=30	↑ *Adlercreutzia, Allobaculum, Escherichia-Shigella, Ileibacterium*, amino sugar & nucleotide sugar metabolism ↓ Gut microbiota count	Mice: ↑ IL-22, COX4, PR-A, MMP, ovarian function ↓ BW, INS, LH, T, mitophagy FMT: recapitulated effects; IL-22 inhibitor: blocked effects Clinical: ↓ IL-22, PR-A, FSH, HDL-C ↑ T, LH, INS, TG, LDL-C, IL-6, IL-1β
Kaur et al. (108)	2022	PCOS=104 (probiotic=52, placebo=52)	↑ 7 probiotic strains (Lactobacillus spp., Bifidobacterium). ↓ Plasma LPS	↑ Menstrual regularity, QoL (menstrual domain); ↓ Total testosterone, WC, WHR; → DHEAS, LH/FSH, FBS, insulin, HOMA-IR, weight, HC, BMI
Szydłowska et al. (109)	2025	PCOS=50 (probiotic=25, placebo=25)	Supplemented 9 probiotic strains (*Bifidobacterium* and *Lactobacillus*)	↓ LH, TSH, androstenedione, BMI ↑ SHBG
Gholizadeh Shamasbi et al. (111)	2018	PCOS=62 (RD = 31, PL = 31)	Resistant dextrin as prebiotic, ↑ Lactobacillus, Bifidobacterium, ↑ SCFAs production	↓ LDL-C, TG, TC, FBS, hsCRP, DHEA-S, free testosterone, hirsutism score, menstrual cycle interval; ↑ HDL-C;
Esmaeilinezhad et al. (112)	2018	PCOS=86 (SPJ = 23, PJ = 23, SB = 23, PL = 23)	Synbiotic (inulin + Lactobacillus) as prebiotic/probiotic, ↑ Lactobacillus, ↑ SCFAs production	↓ HOMA-IR, insulin, BMI, weight, WC, waist/hip ratio, testosterone ↑ QUICKI
Zhou et al. (115)	2023	Clinical: PCOS = 14, Con=8; Rats: CON = 6, PCOS = 6, FMT = 6	PCOS: Dysbiosis (↑ F/B ratio, ↓ α-diversity, altered Lactobacillus, Ruminococcus, Coprococcus, Akkermansia, Desulfovibrio) FMT: Restored microbiota	Rats: ↓ BW, testosterone, cystic follicles, LH/FSH, steroidogenic genes; ↑ Corpora lutea, estrous cycles; FMT reversed changes Clinical: ↑ Testosterone, LH, LH/FSH; ↓ Estradiol

↑ indicates increase/upregulation and ↓ indicates decrease/downregulation.

### 6.1 Dietary interventions

Unhealthy dietary patterns, compounded by hyperandrogenism, hyperinsulinemia, and chronic low-grade inflammation, are widely recognized as metabolic risk factors associated with PCOS (93). A meta-analysis revealed that women with PCOS tend to consume insufficient dietary fiber (94), whereas adequate fiber intake has been demonstrated to reduce body fat and improve glucose metabolism in this population (95). Consequently, dietary intervention, as a cornerstone of lifestyle modification, is recommended as first-line therapy for all women with PCOS (96, 97). Among dietary fibers, inulin has been extensively studied for its beneficial effects on various chronic metabolic disorders, with a recommended daily intake of 5–15 g for adults (98). Geng et al.(2025) demonstrated in an animal study that inulin ameliorates metabolic disturbances and ovarian dysfunction in PCOS rats by modulating the gut microbiota, likely through mechanisms involving enhanced SCFA production and reduced systemic inflammation (99).

While debate persists regarding the optimal dietary pattern for PCOS, the Mediterranean diet (MD), characterized by its richness in unsaturated fats, low-glycemic-index carbohydrates, fiber, and antioxidants, has emerged as a promising therapeutic approach (66). Barrea et al.(2019) observed an inverse correlation between adherence to the MD and PCOS severity, suggesting its potential to mitigate inflammation, insulin resistance, and hyperandrogenism (100). Subsequent studies have indicated that key components of the MD, such as omega-3 fatty acids, antioxidants, and dietary fiber, may reduce inflammation of PCOS through distinct mechanisms (101). In contrast, the ketogenic diet has also shown preliminary promise; studies suggest that after a very low-calorie ketogenic diet (VLCKD), PCOS patients experienced significant improvements in body weight, body composition, metabolic markers (blood glucose, serum insulin, triglycerides, total cholesterol, and LDL cholesterol), as well as insulin resistance (102). Although the VLCKD is an effective short-term therapy for PCOS, this dietary regimen could become unsustainable due to the important restrictions required for ketosis development (103). Thus, no single dietary regimen has been scientifically validated as the gold standard for PCOS management. Future high-quality studies are warranted to elucidate the precise mechanisms and long-term efficacy of diverse nutritional strategies.

### 6.2 Probiotics/prebiotics

Systematic reviews and meta-analyses have demonstrated promising potential for probiotic interventions in PCOS management (104). Probiotic supplementation may improve body weight, BMI, fasting glucose, insulin resistance (HOMA-IR), lipid profiles (triglycerides and very low-density lipoprotein (VLDL)-cholesterol), inflammatory markers such as C-reactive protein (CRP), and oxidative stress indicators including malondialdehyde (MDA), total antioxidant capacity (TAC), and glutathione (GSH), and hormonal parameters (total testosterone, SHBG), while also alleviating hirsutism in PCOS patients (105, 106). Animal studies have further elucidated the molecular mechanisms of specific probiotic strains. Luo et al.(2023) revealed in a DHEA-induced PCOS mouse model that *Escherichia coli* Nissle 1917 could reshape the gut microbiota (e.g., increasing the abundance of *Adlercreutzia* and *Ileibacterium*), thereby promoting IL-22 secretion, improving granulosa cell mitochondrial function (e.g., upregulating cytochrome c oxidase subunit 4(COX4) expression and reducing autophagosome formation), and restoring ovarian hormone balance (107).

To further validate the synergistic effects of multi-strain probiotics, a randomized double-blind placebo-controlled trial involving 104 women with PCOS was conducted over six months, employing a formulation combining seven probiotic strains (*Lactobacillus acidophilus* UBLA-34, *Lactobacillus rhamnosus* UBLR-58, etc.) with fructooligosaccharides, alongside dietary and lifestyle modifications. The study, with its rigorous clinical design, confirmed that the intervention group exhibited significant improvements compared to the placebo group in menstrual cycle regularity (p=0.023), total testosterone levels (p=0.043), waist circumference (p=0.030), and quality of life (p=0.034), with no reported adverse effects (108). Similarly, another 12-week randomized controlled trial using a multi-strain probiotic (SanProbi Barrier) containing nine Lactobacillus and Bifidobacterium strains demonstrated notable improvements in thyroid-stimulating hormone (TSH), androstenedione, SHBG, and BMI among PCOS patients, particularly in those with high free androgen index (FAI) index phenotypes (109).

Notably, the mechanisms of probiotics may involve modulation of gut microbiota composition, while prebiotics such as inulin-type fructans (ITF) can selectively promote the growth of beneficial bacteria like Bifidobacterium and Lactobacillus, enhancing SCFA production and subsequently improving gut barrier function, insulin sensitivity, and metabolic parameters (99, 110). In a 12-week study involving 62 women with PCOS aged 18–45, Gholizadeh et al.(2019) found that daily consumption of 20 g of resistant dextrin led to significant improvements in DHEA levels, free testosterone, and hirsutism scores (111). In contrast, Esmaeilinezhad et al.(2019) reported a significant reduction in total testosterone, but not in LH or FSH, after administration of a synbiotic pomegranate juice containing 20 g of inulin per liter and 2 × 10^8^ colony-forming unit (CFU)/g of Lactobacillus (112). These studies provide evidence-based support for the application of probiotics and prebiotics in the comprehensive management of PCOS, though further exploration is needed to determine long-term efficacy and optimal formulations.

### 6.3 Fecal microbiota transplantation

FMT, an emerging microbial-based therapeutic strategy, involves the transfer of fecal microbial communities from healthy donors into the intestinal tract of patients to restore gut microbiota homeostasis (113). In the treatment of PCOS, FMT is regarded as one of the most promising microbial interventions due to its capacity to comprehensively reshape the gut ecosystem (114). FMT from inulin-treated PCOS patients ameliorated key disorder in antibiotic-treated mice, enhancing insulin sensitivity, reducing lipid accumulation and hyperandrogenism, and improving thermogenesis and ovarian inflammation (99). Similarly, Zhou et al.(2023) demonstrated through systematic animal experiments that FMT not only significantly ameliorated hallmark PCOS symptoms in rats, such as disrupted estrous cycles, elevated testosterone levels, and polycystic ovarian morphology, but also effectively restored gut microbiota diversity, particularly normalizing the abundance of key genera like *Ruminococcus* and *Lactobacillus (*115). Another study by Guo et al. (2016) further confirmed that FMT intervention markedly altered the gut microbiota composition in PCOS rats, characterized by restored abundances of beneficial bacteria such as *Lactobacillus* and *Clostridium* to near-control levels, alongside a reduction in *Prevotella*. These microbial changes were accompanied by improved estrous cyclicity and significantly reduced androgen levels (though not fully normalized) (59). However, direct experimental evidence supporting FMT for PCOS remains limited, with studies predominantly confined to animal models, and no reported human clinical trials to date. Thus, there is an urgent need for prospective clinical research to systematically evaluate the safety, efficacy, and long-term effects of FMT in PCOS patients, facilitating the translation of this innovative therapy from laboratory research to clinical application.

## 7 Discussion

Accumulating evidence suggests that gut microbiome dysbiosis is closely associated with the pathophysiology of polycystic ovary syndrome (PCOS). Alterations in microbial communities and their metabolites have been linked to key pathological features of PCOS (116, 117), including insulin resistance, hyperandrogenism, and chronic low-grade inflammation, supporting the concept of a gut–metabolism–reproductive axis in which intestinal microbial ecosystems influence both metabolic and endocrine homeostasis (23, 73). These findings provide important insights into the mechanisms underlying PCOS and highlight the potential of the gut microbiome as a therapeutic target (104).

Nevertheless, several limitations remain in the current body of research. Considerable heterogeneity exists across studies with respect to microbiome sequencing technologies, bioinformatic analysis pipelines, and population characteristics, which may contribute to inconsistencies in reported microbial signatures. In addition, many available studies rely on relatively small cohorts or cross-sectional designs, limiting the ability to establish causal relationships between gut dysbiosis and PCOS development (61, 72). The functional mechanisms linking specific microbial taxa or microbial-derived metabolites to endocrine and metabolic disturbances also remain incompletely understood. Despite these challenges, growing evidence suggests that microbiome-associated metabolic pathways, including bile acid metabolism, short-chain fatty acid production, and tryptophan metabolism, may represent promising targets for therapeutic intervention (118). Future research integrating multi-omics approaches, such as metagenomics, metabolomics, and host transcriptomics, together with large-scale longitudinal studies and well-designed clinical trials, will be essential to clarify causal mechanisms and evaluate the long-term efficacy of microbiome-targeted interventions. Such advances may ultimately support the development of microbiome-based precision medicine strategies for PCOS management (119, 120).

### 7.1 Future implications

Despite the growing body of evidence linking gut microbiome dysbiosis to the pathophysiology of PCOS, several important questions remain unresolved. Future studies should prioritize well-designed longitudinal and mechanistic investigations to clarify the causal relationships between microbial alterations and key clinical features of PCOS (61, 121). In particular, multi-omics approaches integrating metagenomics, metabolomics, and host transcriptomics may help elucidate the complex interactions between microbial metabolites and endocrine–metabolic pathways. Furthermore, large-scale clinical trials are needed to evaluate the efficacy and safety of microbiome-targeted interventions, such as probiotics, prebiotics, dietary modulation, and fecal microbiota transplantation (104). Advancing our understanding of these mechanisms may ultimately facilitate the development of personalized microbiome-based therapeutic strategies for PCOS management.

## 8 Conclusion

Gut dysbiosis plays a pivotal role in the pathogenesis of metabolic dysfunction-associated PCOS, with microbial imbalance closely linked to hyperandrogenemia, insulin resistance, and ovulatory dysfunction. A deeper understanding of the mechanistic interplay between the gut microbiome and PCOS paves the way for developing targeted microbiota-modulating therapies—such as probiotics, fecal microbiota transplantation and dietary modifications—to restore gut ecological equilibrium. Future research should further elucidate the regulatory roles of microbial metabolic networks (e.g., bile acids, SCFAs, and tryptophan metabolites) and leverage multi-omics technologies to refine personalized treatment strategies, thereby distinguishing therapeutic responses across PCOS subtypes and advancing precision medicine in reproductive endocrinology.

## Glossary

AhR: aryl hydrocarbon receptor
AMH: anti-Müllerian hormone
AUC: area under the curve
BMI: body mass index
BSH: bile salt hydrolase
CA: cholic acid
CDCA: chenodeoxycholic acid
CFU: colony-forming unit
CLGI: chronic low-grade inflammation
COX4: cytochrome c oxidase subunit 4
CRP: C-reactive protein
cAMP: cyclic adenosine monophosphate
DCA: deoxycholic acid
DHEA: dehydroepiandrosterone
FAI: free androgen index
FFAR2 (GPR43): free fatty acid receptor 2
FFAR3 (GPR41): free fatty acid receptor 3
FFAs: free fatty acids
FMT: fecal microbiota transplantation
FSH: follicle-stimulating hormone
FXR: farnesoid X receptor
GABA: γ-aminobutyric acid
GDCA: glycodeoxycholic acid
GLP-1: glucagon-like peptide-1
GnRH: gonadotropin-releasing hormone
GnRHR: gonadotropin-releasing hormone receptor
GSH: glutathione
HDAC: histone deacetylase
HFD: high-fat diet
HO-1: heme oxygenase-1
HPO axis: hypothalamic–pituitary–ovarian axis
IAA: indole-3-acetic acid
IDO1: indoleamine 2,3-dioxygenase 1
IFN-γ: interferon-γ
IL-1β: interleukin-1 beta
IL-6: interleukin-6
IL-10: interleukin-10
IL-17: interleukin-17
IL-22: interleukin-22
ILC3s: group 3 innate lymphoid cells
IR: insulin resistance
IR-PCOS: insulin-resistant polycystic ovary syndrome
IRS-1: insulin receptor substrate-1
ITS: internal transcribed spacer
ITF: inulin-type fructans
JNK: c-Jun N-terminal kinase
KEGG: Kyoto Encyclopedia of Genes and Genomes
Kiss1-GPR54-PKC-ERK1/2: Kisspeptin-G protein-coupled receptor 54-protein kinase C-extracellular signal-regulated kinases 1/2 signaling pathway
LH: luteinizing hormone
LPS: lipopolysaccharide
MDA: malondialdehyde
MD: Mediterranean diet
mRNA: messenger RNA
MnSOD: manganese superoxide dismutase
MyD88: myeloid differentiation primary response 88
NLRP3: NLR family pyrin domain containing 3
NOX2/4: NADPH oxidase 2/4
Nrf2: nuclear factor erythroid 2-related factor 2
PCOS: polycystic ovary syndrome
PD: phylogenetic diversity
PI3K: phosphoinositide 3-kinase
PYY: peptide YY
ROS: reactive oxygen species
SCFAs: short-chain fatty acids
SHBG: sex hormone-binding globulin
SOCS3: suppressor of cytokine signaling 3
TAC: total antioxidant capacity
TGR5: Takeda G protein-coupled receptor 5
TICs: theca-interstitial cells
TLRs: Toll-like receptors
TLR4: Toll-like receptor 4
TMAO: trimethylamine-N-oxide
TNF-α: tumor necrosis factor-α
TSH: thyroid-stimulating hormone
TUDCA: tauroursodeoxycholic acid
VLCKD: very low-calorie ketogenic diet
VLDL: very low-density lipoprotein
vOTU: viral operational taxonomic unit
ZO-1: Zonula occludens-1
3β-HSD: 3β-hydroxysteroid dehydrogenase
5-HT: 5-hydroxytryptamine.
